# Incidental Intracranial Findings and Their Clinical Impact; The HUNT MRI Study in a General Population of 1006 Participants between 50-66 Years

**DOI:** 10.1371/journal.pone.0151080

**Published:** 2016-03-07

**Authors:** Asta Kristine Håberg, Tommy Arild Hammer, Kjell Arne Kvistad, Jana Rydland, Tomm B. Müller, Live Eikenes, Mari Gårseth, Lars Jacob Stovner

**Affiliations:** 1 Department of Neuroscience, Norwegian University of Science and Technology (NTNU), Trondheim, Norway; 2 Department of Radiology, St. Olav University Hospital, Trondheim, Norway; 3 Department of Neurosurgery, St. Olav University Hospital, Trondheim, Norway; 4 Department of Circulation and Medical Imaging, NTNU, Trondheim, Norway; 5 Department of Radiology, Levanger Hospital, Levanger, Trondheim, Norway; 6 Department of Neurology and Clinical Neurophysiology, St. Olav University Hospital, Trondheim, Norway; University of Manchester, UNITED KINGDOM

## Abstract

**Objectives:**

Evaluate types and prevalence of all, incidental, and clinically relevant incidental intracranial findings, i.e. those referred to primary physician or clinical specialist, in a cohort between 50 and 66 years from the Nord-Trøndelag Health (HUNT) study. Types of follow-up, outcome of repeated neuroimaging and neurosurgical treatment were assessed.

**Material and Methods:**

1006 participants (530 women) underwent MRI of the head at 1.5T consisting of T1 weighted sagittal IR-FSPGR volume, axial T2 weighted, gradient echo T2* weighted and FLAIR sequences plus time of flight cerebral angiography covering the circle of Willis. The nature of a finding and if it was incidental were determined from previous radiological examinations, patient records, phone interview, and/or additional neuroimaging. Handling and outcome of the clinically relevant incidental findings were prospectively recorded. True and false positives were estimated from the repeated neuroimaging.

**Results:**

Prevalence of any intracranial finding was 32.7%. Incidental intracranial findings were present in 27.1% and clinically relevant findings in 15.1% of the participants in the HUNT MRI cohort. 185 individuals (18.4%) were contacted by phone about their findings. 40 participants (6.2%) underwent ≥ 1 additional neuroimaging session to establish etiology. Most false positives were linked to an initial diagnosis of suspected glioma, and overall positive predictive value of initial MRI was 0.90 across different diagnoses. 90.8% of the clinically relevant incidental findings were developmental and acquired cerebrovascular pathologies, the remaining 9.2% were intracranial tumors, of which extra-axial tumors predominated. In total, 3.9% of the participants were referred to a clinical specialist, and 11.7% to their primary physician. 1.4% underwent neurosurgery/radiotherapy, and 1 (0.1%) experienced a procedure related postoperative deficit.

**Conclusions:**

In a general population between 50 and 66 years most intracranial findings on MRI were incidental, and >15% of the cohort was referred to clinical-follow up. Hence good routines for handling of findings need to be in place to ensure timely and appropriate handling.

## Introduction

Cerebral magnetic resonance imaging (MRI) is increasingly used in the clinic due to its superior ability to visualize and differentiate between brain pathologies. It is also used with increasing frequency in studies of neurological, neurosurgical and psychiatric diseases and disorders [1–3], brain development and aging [4–6] and neurobiology [7, 8]. Cerebral MRI obtained for clinical or research purposes can uncover incidental, i.e. unexpected, findings. It is important to know both the prevalence of incidental findings in the brain and the clinical consequences, including outcome, after discovery of such findings in unselected populations. The prevalence of incidental findings on MRI of the head has been investigated in several studies with inconsistent results, indicating that MRI methodology (e.g. scan protocol, hardware, reading) and population selection (e.g. hospital samples, age range and ethnicity) have significant impact on discovering incidental intracranial findings [9–13]. However, whether a finding is truly incidental or represents pathology previously described clinically or radiologically has not been systematically investigated. Moreover, the clinical consequences of uncovering incidental findings in terms of referral to different kinds of follow-up, has been investigated in only a few studies [10, 13–16]. No study has followed up on types of referral and outcome after procedures related to uncovering incidental intracranial findings in the course of a research study. The prevalence of different types of intracranial findings, whether a finding is incidental, its clinical impact and the rate of a false positives, as well as the types of follow-up needed to treat an incidental finding according to established guidelines, have significant administrative, logistic and health economic implications for the study organizers, and may also have life altering and economic consequences for MRI study participants.

The current study was designed to fill the knowledge gap concerning the prevalence of incidental intracranial findings, false positives on initial brain MRI of suspected neurosurgical conditions, the clinical consequences of uncovering incidental intracranial findings, i.e. additional neuroradiological procedures, referral to primary physician or clinical specialists, and the outcome after neurosurgical/radiotherapy intervention.

## Material and Methods

The study was approved by the HUNT study board of directors and the Helse Midt-Norge regional ethics and health research committee, REK midt (2011/456). All participants were adults and legally competent and gave their informed written consent, which is kept in a fireproof safe in a locked room.

### The HUNT population and the HUNT MRI cohort

The Nord-Trøndelag Health Study (HUNT) is a large multiphase, multipurpose health study on the inhabitants ≥ 13 years in the county of Nord-Trøndelag, Norway [17, 18]. In the first wave of HUNT in 1984–1986 (HUNT 1) the entire county’s inhabitants ≥ 20 years were invited to participate and the response rate was 88.1%. The participants filled in two questionnaires, clinical measures were obtained and blood samples drawn. The second survey, HUNT 2 took place in 1995–1997 and was more comprehensive. In this survey, all inhabitants ≥ 13 years were invited, and the survey was split into two groups, young-HUNT (age 13–19) and adult-HUNT (≥ 20 years). The overall response rate was 70%. The adult participants were subjected to a comprehensive evaluation with questionnaires, clinical measures and blood samples. A third survey (HUNT 3) took place from 2006–2008, where all inhabitants ≥ 13 years were invited. For the cohort aged 50–69, roughly the age group invited to participate in HUNT MRI, the response rate was 67.6%, increasing with age and higher in women, where the response rate was 74.5% in the 60–69 year group. The cohort invited to participate in HUNT MRI was drawn from the HUNT population, but limited to volunteers who had participated in HUNT 1, 2 and 3, and were between 50–65 years at time of inclusion. This age range and participation in HUNT1, 2 and 3 were chosen for several reasons; to ensure that all participants had longitudinal questionnaire, clinical and blood work data, the age range coincides with the time when age-related pathologies start to emerge, such as cerebrovascular disease and tumors [12, 15], but is before onset of the common dementias [19, 20], and the age range overlaps with the median age of those referred to clinical brain MRIs in our clinic.

**Fig 1:**
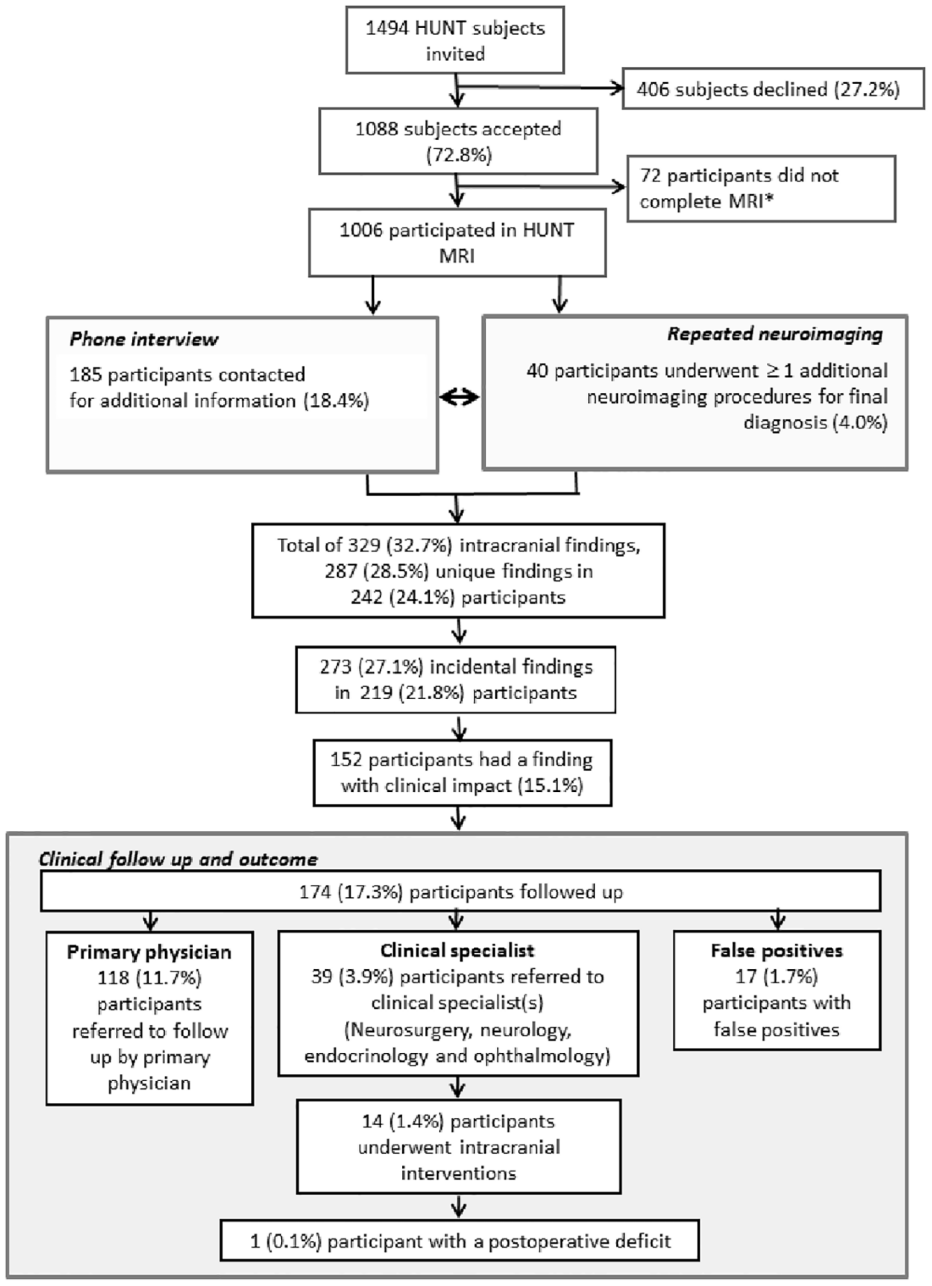
Flow diagram and overview of inclusion and exclusion of participants in the HUNT MRI study, the types and prevalence of classes of intracranial findings, follow up and outcome in the included participants.

In the HUNT MRI study the goal was to include 1000 subjects who had participated in HUNT1, 2 and 3, and was between 50–65 years with an equal sex and age distribution across the age range. For practical reasons a further inclusion criterion was living within 45 minutes driving distance from Levanger Hospital where the MRI scanning was performed. Exclusion criteria were limited to the usual MRI contraindications, including weight >150 kg. In total 1560 individuals from the HUNT cohort who fulfilled the inclusion criteria were selected from the HUNT population. To obtain the desired sex and age distribution within the group, 66 of the 1560 persons fulfilling the inclusion criteria were not invited to participate, leaving 1494 to be invited. See Fig 1 for flow chart of participant inclusion and outcomes.

In total 406 subjects (27.2%) declined the invitation or did not answer while 1088 (72.8%) subjects gave informed consent. Of the included participants, 1006 had successful MRI scans. Reasons for unsuccessful MRIs were termination of scanning due to claustrophobia (n = 16), muscle cramps (n = 5), unsuccessful image acquisition due to metallic artifacts (n = 3), last minute cancellation (n = 28), did not show up (n = 5), contraindications uncovered by the MRI technologist before scanning (n = 4), moved (n = 1), died (n = 1), was >65 years (n = 1) or hospitalized (n = 1). When the number of participants surpassed 1000, MRI data collection was closed and the planned scanning of the remaining 17 consenting individuals cancelled. For details on health and clinical measures plus socioeconomic demographics in the subjects included in HUNT MRI compared to all HUNT participants in the same age range and to those declining participation see [21]. In general, the HUNT MRI participants were very similar to non-participants and the non-invited HUNT participants [22]. In comparison to both non-participants and non-invited HUNT subjects, the HUNT MRI participants had a higher level of education already at time of the first survey, HUNT 1, considered to be due to the inclusion criteria of living ≤ 45 min driving distance from the hospital, i.e. less rural area than most of the county. Furthermore, HUNT MRI participants had lower body mass index, due to weight ≥ 150 kg being a MRI exclusion criterion, and also a somewhat better cardiovascular disease risk profile. As of time of submitting the manuscript, none of the HUNT MRI participants have been diagnosed with mild cognitive impairment or dementia.

### MRI scan protocol

The MRI exanimation took ~50 minutes for each participant and the first examination took place on the 21^st^ of July 2007, and the last examination on the 10^th^ of December 2009.

**Fig 2:**
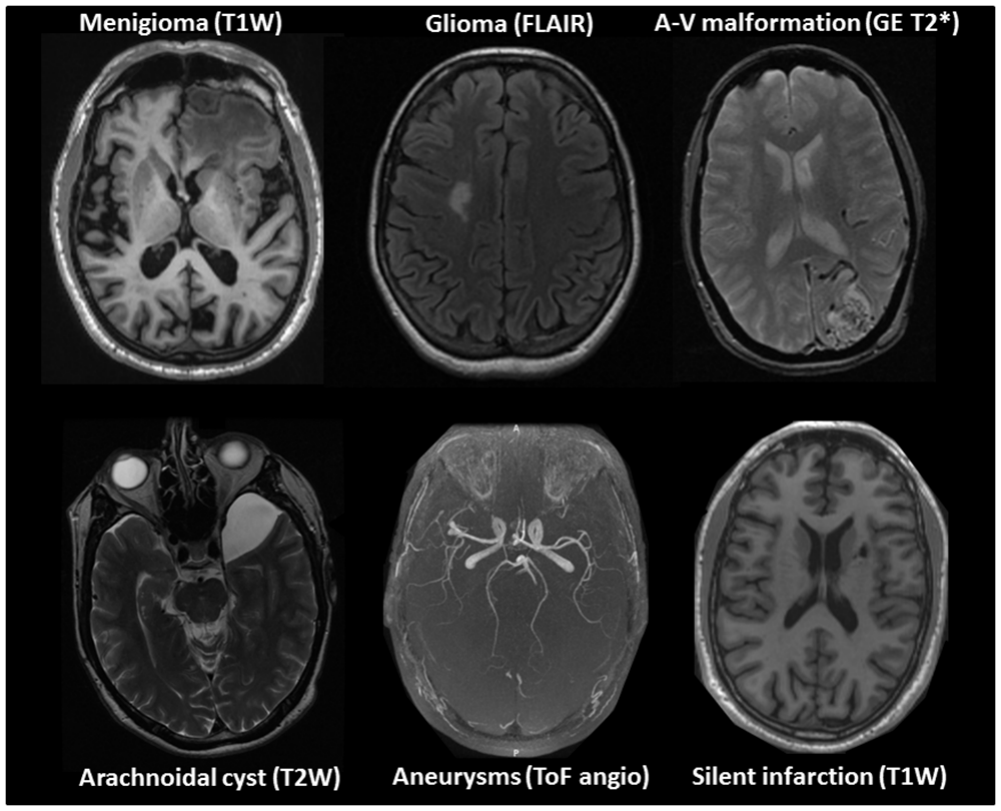
Examples of the different scan sequences and incidental intracranial findings in the HUNT MRI cohort, i.e. meningioma (T1W 3D), glioma (FLAIR), arteriovenous malformation (T2* weighted), arachnoidal cyst (T2W weighted), aneurysms (Time of Flight angio) and silent infarctions (T1W 3D scan).

All imaging was performed on the same 1.5 T General Electric Signa HDx 1.5 T MRI scanner equipped with an eight channel head coil (GE Healthcare) and software version pre-14.0M4. The examinations were conducted by eight MRI technologists following a standardized written and illustrated procedure. All volunteers underwent the same scan protocol. The MRI protocol included a sagittal T1 weighted IR-FSPGR volume, the Alzheimer’s disease Neuroimaging Initiate (ADNI) volume, (http://adni.loni.usc.edu/methods/documents/mri-protocols/), axial T2-, T2*-weighted and FLAIR sequences obtained parallel to the anterior-posterior commissure line, and a time of flight (TOF) 3D angio sequence angled to cover the entire Circle of Willis. See Table 1 for scan sequence parameters, and Fig 2 for examples of the different MRI sequences. In addition, diffusion tensor imaging was performed, but this sequence was not evaluated as part of the current study.

**Table 1:** Scan parameters for the different sequences in HUNT MRI.

MRI sequence	Matrix size	NSA	TR (ms)	TE (ms)	Flip-angle	Slice thickness (mm)	Gap (mm)	Overlap (mm)	FOV (mm)
**IR-FSPGR**	192x192	1	10.2	4.1	10°	1.2	0	0	240
**T2W**	512x320	2	7840.0	95.3	90°	4.0	1	0	230
T2* W	256x192	1	500.0	20.0	20°	4.0	1	0	230
**FLAIR**	256x224	1	11,002.0	122.9	90°	4.0	1	0	230
**TOF-angio 3D**	320x224	1	24.0	2.7	20°	1.0	0	5.0	200

All imaging was performed on the same 1.5 T General Electric Signa HDx 1.5 T magnetic resonance imaging (MRI) scanner equipped with an eight channel head coil and software version pre-14.0M4. **IR-FSPGR, inversion recovery prepared fast spoiled grass**; GRE, gradient echo; T2W, T2 weighted; T2*W, T2* weighted; FLAIR; fluid attenuated inversion recovery; ToF-angio, Time of flight angiography; NSA, number of signal averages; TR, repletion time; TE, Echo time; FOV, field of view.

### Assessment of images and handling of findings

**Fig 3:**
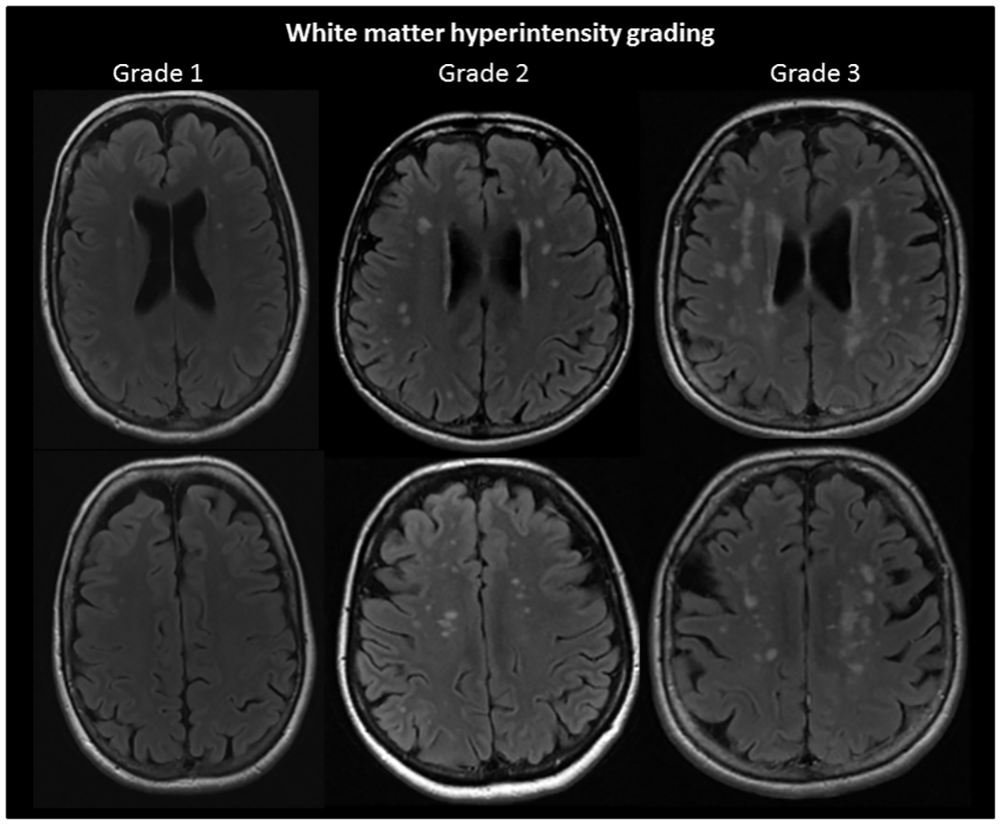
Typical examples of Fazekas grade 1, 2 and 3 white matter hyperintensities (WMH). The examples are from the FLAIR scans of three HUNT MRI participants.

Image readings were performed using a standard clinical digital picture archiving and communications system. All images were assessed independently by two experienced senior neuroradiologists (KAK and JR). The different intracranial findings were described based on standard neuroradiological procedures, including repeated neuroimaging (see below). Calcifications were determined based on T2* scans in combination with the other MRI scans. For white matter hyperintensities (WMH), a modified Fazekas score was used to classifying the lesions into grade 0–3, see Fig 3 [23–25]. Based on previous studies, WMH scores grade 0 and 1 were considered normal in this age group while grades 2 and 3 were classified as excessive [24]. Subjects with multiple sclerosis (n = 3) were excluded in the analysis of the prevalence of excessive WMH. Excessive WMH is recognized as having negative consequences for an individual’s future cognitive health, independence and survival [26–28]. Intervention, in particular treating hypertension [27], appears to modify the development and hence also negative consequences of increasing load of WMH. Hence, Fazekas score ≥2 was deemed clinically relevant.

The neuroradiologists were not provided with clinical information on earlier findings when first reading the images, but were not blinded to the volunteer’s name, birth and examination date. In case of a finding the radiologists were able to access all earlier radiological examinations and patient records. In cases of uncertain findings repeated neuroimaging was performed. Modality and sequences used depended on the clinical question and included MRI with or without contrast agent, MR spectroscopy, CT and/or angiography. All the available HUNT MRI data, and in certain cases pre-existing CT or MRI data, and/or follow-up MRI/MRS, CT or angiography were used to determine the presence and nature of a finding. In this paper only intracranial findings are reported, but all findings were described by the neuroradiologists, and followed up clinically if necessary. For the findings in the paranasal sinuses see [29]. After independent evaluation of all images from one subject by both neuroradiologists, consensus was reached, which led to the final report. From the time of acquisition, most examinations were reviewed within 2 weeks and all within 40 days.

All participants received a letter where the result of the MRI examination was described.

The different incidental findings were treated according to standard clinical procedures. The radiological reports were handed over to a senior neurologist (LJS), who contacted participants by phone to inform of a finding, gather additional information on medical history to further classify a finding into e.g. clinical or silent infarctions, participant aware of having excessive WMH or not, and inform about follow-up procedures, i.e. additional neuroimaging, referral to primary physician or clinical specialist. The general practitioners of the participants were informed in writing about finding(s) if deemed clinically relevant and the participant consented. Informing and referring a participant to a clinical specialist and/or follow-up imaging to ascertain a diagnosis did not require consent from the participants as outlined in the consent form and reflected in the ethical committee’s mandate. Participants were referred to the appropriate clinical specialist depending on type of incidental finding. In most cases with excessive WMH, the primary physician was contacted if participant consented, but in a few severe cases the participants were referred to a neurologist. Participants referred to the neurosurgical department and/or other clinical specialists were treated according to standard protocols with regard to referral, treatment and follow up. Treatment of aneurysms were based on clinical factors known to influence rupture risk [30] in accordance with suggested guidelines [31]. For those undergoing neurosurgery due to incidental findings on MRI in connection to HUNT MRI, all peri- and postoperative complications were recorded.

### Categorization of findings

Findings were classified into (1) all intracranial findings, i.e. previously described or known plus new findings, (2) incidental intracranial findings; any finding which was not previously described in patient records and/or was unknown to the participant, and/or not fitting clinical information obtained from participant via phone interview, and (3) incidental intracranial findings with clinical impact; incidental findings leading to referral to clinical specialist or primary physician, and/or surgery or another type of intervention (e.g. radiation therapy). All follow up neuroimaging performed in order to verify a tentative diagnosis based on the initial MRI was recorded. Follow-up neuroimaging was performed in cases with a finding potentially in need of neurosurgical treatment based on the initial MRI. From the follow-up imaging data, false positives were registered, but not false negatives which were beyond the scope of the present study. The reported findings are the final diagnosis after follow-up MR/CT/angiography and/or other diagnostic procedures, information from participants’ hospital records and radiological reports plus phone interview. More than one type of finding, and/or the presence of more than one lesion of the same type sometimes occurred in the same participant.

### Statistical analysis

All data analysis was done in IBM SPSS Statistics 22 (SPSS Inc., Chicago, IL, USA). The following was calculated for all intracranial findings: total number, prevalence (%) with 95% confidence interval (CI); for all intracranial finding per individual: total number, prevalence (%) with 95% CI, and finally the ratio of men to women having each type of finding. The findings were subsequently divided into incidental intracranial findings and incidental intracranial findings with clinical impact. Numbers of true and false positives (overall and by diagnosis) were calculated based on the follow up neuroimaging data. Positive predictive value of initial MRI was calculated. The effect of sex on prevalence of the different intracranial findings was investigated. The effect of age was assessed for strokes and presence of age appropriate versus excessive WMH. Differences between groups were analyzed with an unpaired t-test, and between proportions with Fisher’s exact test.

The statistical significance was set to *p* < 0.05, two-tailed. Effect sizes were calculated with Cohen’s *d*. Results are given as number of, percentage with 95% CI where appropriate, male: female ratio, and as mean ± standard deviation for group data.

## Results

**Fig 4:**
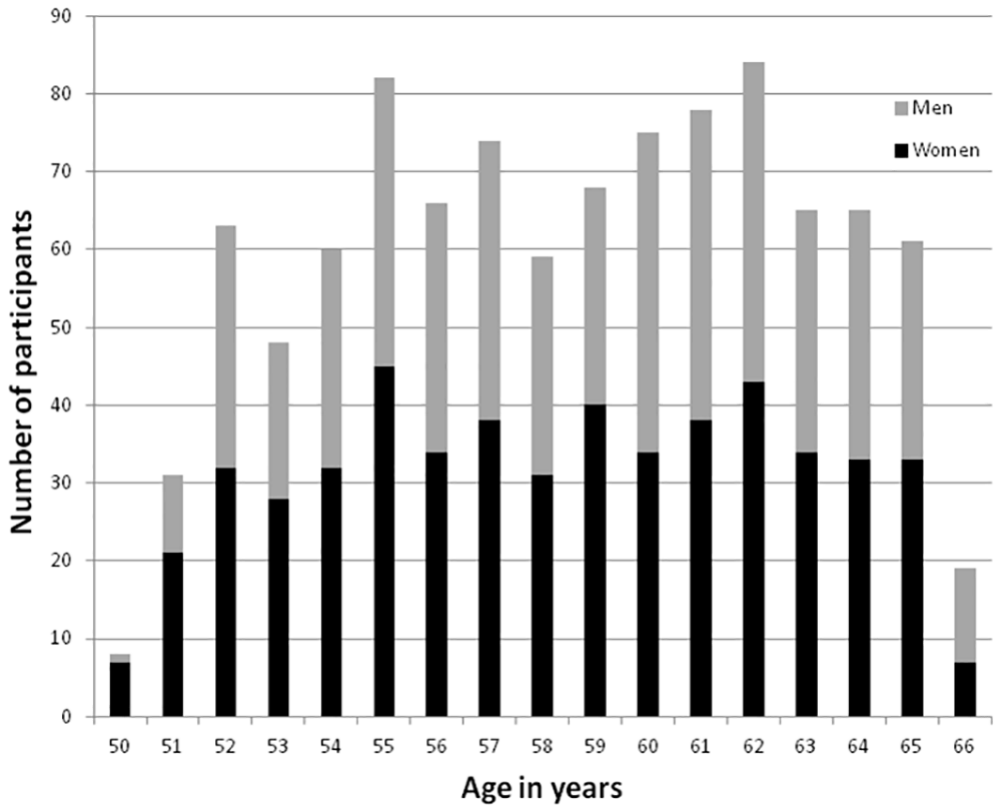
Age and sex distribution in the HUNT MRI cohort.

In HUNT MRI 1006 successful MRI examinations were performed on 476 males (47.3%) and 530 females (52.7%), with a median age of 59.2 ± 4.2 years and a range between 50.5 and 66.8 years (Figs 1 and 4). A delay between time of inclusion and the MRI scan led to fewer participants being 50 years of age, and that 19 individuals were 66 years because they had their birthday between the time of consent and the MRI examination.

See Fig 1 for a comprehensive overview of participant invitation, inclusion, radiological and clinical follow up and outcome in the HUNT MRI study. In total 185 participants (18.4%, 95% CI 16–21%) were contacted concerning intracranial findings in order to obtain medical history and other relevant information, and/or inform the subject about finding(s) and referral to additional neuroimaging, clinical specialist or primary physician.

### Prevalence of intracranial findings

A total of 329 different intracranial findings were detected giving an overall prevalence of a finding of 32.7% (Table 2, Fig 1). When counting several of the same type of finding as one finding (e.g. several aneurysms or contusion) in the same subject, a total of 287 unique types of findings were present (Table 2, Fig 1). Thus, overall prevalence for each specific intracranial finding per participant was 28.5% (Table 2, Fig 1). These findings were detected in 242 of the 1006 participants (24.1%, 95% CI 22–27%), equally distributed among 133 women (55.0%) and 109 men (45.0%) (Table 2). In 44 individuals (18.1% of those with any findings, 95% CI 14–23%), ≥ 2 different types of findings were present. The most frequent combinations of findings were excessive WMH combined with stroke (n = 9), cysts (n = 4), or aneurysms (n = 2). All other combinations were one of a kind.

**Table 2:** Number and prevalence intracranial findings stratified into total number of findings, participants with findings, distribution of findings between men and women, total incidental findings, and number of individuals with incidental findings with clinical impact in the HUNT-MRI cohort of 1006 participants (476 men and 530 women).

Type of Finding	Total number of each finding with (%) and [95% CI]	Number of individuals with each type of finding with (%) and [95% CI]	Total number of men: women with each type of finding	Total number of incidental findings with (%) and [95% CI]	Number of individuals with findings of clinical impact^#^ with (%) and [95% CI]
***Cysts***					
Arachnoid cyst	36 (3.6%) [2–5%]	36 (3.6%) [2–5%]	21:15	36 (3.6%) [2–5%]	0
Other cysts^1^	13 (1.3%) [1–2%]	13 (1.3%) [1–2%]	3:10	13 (1.3%) [1–2%]	0
***Structural vascular abnormality***					
Cerebral aneurysm	23 (2.3%) [1–3%]	19 (1.9%) [1–3%]	5:14	23 (2.3%)[1–3%]	19 (1.9%) [1–3%]
Cavernous hemangioma	3 (0.3%) [0–1%]	3 (0.3%) [0–1%]	1:2	3 (0.3%) [0–1%]	1 (0.1%) [0–0%]
Deep venous anomaly	4 (0.4%) [0–1%]	4 (0.4%) [0–1%]	2:2	4 (0.4%) [0–1%]	0
AV^2^ malformation	1 (0.1%) [0–0%]	1 (0.1%) [0–0%]	0:1	1 (0.1%) [0–0%]	1 (0.1%) [0–0%]
Stenosis of middle cerebral artery	2 (0.2%) [0–0%]	2 (0.2%) [0–0%]	1:1	0	0
Occlusion internal carotid	5 (0.5%) [0–1%]	5 (0.5%) [0–1%]	3:2	2 (0.2%) [0–0%]	2 (0.2%) [0–0%]
Stenosis of internal carotid artery	1 (0.1%) [0–0%]	1 (0.1%) [0–0%]	0:1	1 (0.1%) [0–0%]	1 (0.1%) [0–0%]
***Other developmental variations***					
Heterotopia	2 (0.2%) [0–0%]	2 (0.2%) [0–0%]	2:0	2 (0.2%) [0–0%]	0
Chiari malformation gr. 1	2 (0.2%) [0–0%]	2 (0.2%) [0–0%]	1:1	2 (0.2%) [0–0%]	0
Megacisterna magna	1 (0.1%) [0–0%]	1 (0.1%)[0–0%]	0:1	1 (0.1%) [0–0%]	0
Septum pellucidum/cavum vergea^3^	7 (0.7%) [0–1%]	7 (0.7%) [0–1%]	5:2	7 (0.7%) [0–1%]	0
***Infarctions***^4^					
Cortical	12 (1.2%) [0–2%]	12 (1.2%) [0–2%]	7:4	3 (0.3%) [0–0%]	3 (0.3%) [0–0%]
Sub-cortical	5 (0.5%) [0–1%]	5 (0.5%) [0–1%]	4:1	2 (0.2%) [0–0%]	2 (0.2%) [0–0%]
Lacunar	27 (2.7%) [2–4%]	19 (1.9%) [1–3%]	11:8	22 (2.2%) [1–3%]	14 (1.4%) [0–2%]
Cerebellar	15 (1.5%) [1–2%]	15 (1.5%) [1–2%]	7:8	10 (1.0%) [0–2%]	10 (1.0%) [0–2%]
***Excessive white matter hyperintensities***^5^	91 (9.1%) [7–10%]	91 (9.1%) [7–10%]	34:57*	84 (8.4%) [6–10%]	84 (8.4%) [6–10%]
***Microhemorrhages***	41 (4.1%) [0–8%]	13 (1.3%) [1–2%]	6:7	41 (4.1%) [0–8%]	1 (0.1%) [0–0%]
***Parenchymal calcification***	2 (0.2%) [0–0%]	2 (0.2%) [0–0%]	0:2	2 (0.2%) [0–0%]	0
***Tumors***					
Glioma	1 (0.1%) [0–0%]	1 (0.1%) [0–0%]	0:1	1 (0.1%) [0–0%]	1(0.1%) [0–0%]
Meningioma	10 (1.0%) [0–2%]	10 (1.0%) [0–2%]	1:9**	10 (1.0%) [0–2%]	10 (1.0%) [0–2%]
Pituitary tumors	3 (0.3%) [0–1%]	3 (0.3%) [0–1%]	1:2	2 (0.2%) [0–0%]	2 (0.2%) [0–0%]
Vestibular schwannoma	1 (0.1%) [0–0%]	1 (0.1%) [0–0%]	1:0	1 (0.1%) [0–0%]	1(0.1%) [0–0%]
***Other acquired brain pathologies***					
Contusions	12 (1.1%) [0–2%]	10 (0.9%) [0–1%]	7:3	0	0
Multiple sclerosis	3 (0.3%) [0–1%]	3 (0.3%) [0–1%]	2:1	0	0
Progressive supranuclear palsy	1 (0.1%) [0–0%]	1 (0.1%) [0–0%]	1:0	0	0
Postoperative changes	5 (0.5%) [0–1%]	5 (0.5%) [0–1%]	2:3	0	0
**Total number (%)**	329 (32.7%) [30–36%]	287 (28.5%) [26–31%]	109 (45.0%): 133 (55.0%)	273 (27.1%) [24–30%]	152 (15.1%) [13–17%]

The findings are categorized based on their finale etiology as determined from HUNT MRI scans, previous medical records and radiological examinations, phone interview, and in some cases repeated neuroimaging. False positives are reported in Table 3.

^#^ Incidental intracranial findings with clinical impact are those leading to referral to primary physician or clinical specialist. Participants referred to follow-up neuroimaging which did not confirm the initial diagnosis or fall into the other categories in this table are not included here, see Table 3 for this information.

^1^ Rathke’s cleft (n = 2), neuroepithelial subcortical (n = 1), ependymal (n = 9), and one pineal gland (n = 1) cysts.

^2^ AV, arteriovenous

^3^ Three participants had septum pellucidum and cavum vergae.

^4^ 44 individuals had one or more strokes.

^5^ Excessive WMH were defined as score ≥ 2 on the modified Fazekas scale (see Methods and Fig 2 for details), excluding those with multiple sclerosis.

* Significant sex-specific difference in prevalence, p = 0.048, two-tailed

** Significant sex-specific difference in prevalence, p = 0.02, two-tailed

### Prevalence of incidental intracranial findings

There were 273 incidental intracranial findings in the HUNT MRI cohort, giving an overall prevalence of incidental findings of 27.1% across the sample (Fig 1 and Table 2). Again, some participants had ≥ 2 incidental intracranial findings, i.e. the incidental findings were present in 219 HUNT MRI participants (21.8%, 95% CI 19–24%). Hence, 83.0% (95% CI 79–87%) of all intracranial findings were incidental. The previously recognized intracranial findings included various acquired brain disorders such as stroke, traumatic contusions and multiple sclerosis (Table 2). In total 152 participants had an incidental intracranial finding with clinical impact, giving a prevalence of 74.4% (95% CI 69–80%) of all incidental intracranial findings being clinically relevant, or 15.1% (95% CI 13–17%) of all HUNT MRI participants having an incidental finding with clinical impact (Table 2 and Fig 1). The incidental findings with clinical impact were in 90.8% (95% CI 86–95%) of the cases related to various acquired and developmental cerebrovascular pathologies (aneurysms, arteriovenous malformation, cavernous hemangioma, occlusion/stenosis of arteries, infarctions, WMH, microhemorrhages). The second largest class of clinically relevant incidental findings was extra- and intra-axial brain tumors (9.2%, 95% CI 4–13%). The bulk was extra-axial tumors, with a ratio of 13 extra-axial to one intra-axial tumor.

In 40 participants (4.0%, 95% CI 0–8%, of the entire cohort), one or more additional neuroimaging procedures was performed to verify or rule out the presence of a suspected intracranial tumor or aneurysm (Fig 1 and Table 3). The original diagnosis was verified in 23 participants, while a less serious differential diagnosis was assigned in 17 cases, giving an overall significantly higher chance of a true positive (p< 0.0001, Table 3). The overall prevalence of false positives in HUNT MRI was 1.7% (95% CI 1–2%), thus the overall positive predictive value of the initial MRI diagnosis was 0.90. Breaking the diagnoses down to specific entities, the predominant reason for a false positive was a suspected glioma, which was demonstrated to be a benign lesion in 93.8% of the cases (95% CI 80–100%) (Table 3). The positive predictive value of the initial MRI for diagnosing a glioma was very poor at 0.06. For aneurysms the positive predictive value of the initial MRI was 0.90. There were no false positives for meningioma, pituitary tumors or vestibular schwannoma, giving a positive predictive value of 1.

**Table 3:** False and true positive intracranial findings and positive predictive value of initial MRI based on follow-up neuroimaging in participants in the HUNT MRI cohort with a suspected neurosurgical condition.

Type of uncertain finding on initial MRI	Total number	Number of true positives with (%)	Number of false positives with (%)	Final classification of false positives
Cerebral aneurysm	11	9 (81.2%)	2 (18.2%)	Artifact (n = 1), Ectasia (n = 1)
Glioma	13	1 (7.7%)	12 (92.3%)	Gliosis/WMH (n = 6), Cyst (n = 2), Benign unspecific lesion^a^ (n = 4)
Meningioma	10	10 (100%)	0 (0%)	NA
Pituitary tumors	2	2 (100%)	0 (0%)	NA
Vestibular schwannoma	1	1 (100%)	0 (0%)	NA
**Total number**	**37**	23 (62.2%)*	**14 (37.8%)**	

^a^ Benign unspecified lesion; a lesion not possible to classify from its MRI characteristics, but considered benign based on ≥ 3 repeated imaging over 2 years. CI, confidence interval; WMH, white matter hyperintensities; NA, not applicable

*p<0.0001 two tailed

Overall, 174 participants (17.3%, 95% CI 15–21%) were followed up with additional neuroimaging and/or clinically (primary physician or specialist). In total, 118 (11.7%, 95% CI 10–14%) HUNT MRI participants agreed to their primary physician being informed about a clinically relevant incidental finding which did not require specialist care. There were 39 (3.9%, 95% CI 3–5%) participants referred to ≥ 1 clinical specialist(s) (Fig 1).

### Referral to neurosurgeon and results of treatment

The 35 participants (3.5%, 95% CI 2–5%) referred to the Department of Neurosurgery had the following findings: aneurysms (n = 19), arteriovenous malformation (n = 1), the largest cavernous hemanigoma (8x4 mm) (n = 1), glioma (n = 1), meningioma (n = 10), vestibular schwannoma (n = 1), and pituitary tumors (n = 2). Overall, 14 individuals (1.4%, 95% CI 0–2%) underwent some type of intracranial intervention to treat a condition detected in HUNT MRI. Nine of the 19 individuals with aneurysms were treated. Three patients experienced procedure related complications, but all recovered without neurological sequelae. The arteriovenous malformation (see Fig 2) was microsurgically removed, and resulted in a postoperative permanent hemi-quandrantopia. The glioma was resected and re-resected three years later. The operations did not result in postoperative neurological deficits, and the participant is still alive 7 years after the diagnosis. Two of the 10 meningiomas were resected without complications. The remaining eight meningiomas are followed up based on standard routines. The vestibular scwhannoma was successfully treated with gamma-knife. The two incidental pituitary tumors are treated conservatively with follow-up. Overall, 14 individuals (1.4%, 95% CI 0–2%) underwent some type of intracranial intervention to treat a condition detected in HUNT MRI. One subject (0.1%, 95% CI 0–0.03%) suffered from a postoperative neurological deficit (Fig 1).

### Overview of prevalence all intracranial findings

#### Cysts

There were 36 (3.6%) arachnoid cysts in 36 individuals (Fig 2), the largest cyst was located in the midline of the posterior fossa and had a diameter of 6 cm. There was no difference in the lateralization of the arachnoid cysts. Nineteen (53%, 95% CI 36–70%) of the arachnoid cysts were located over the hemispheres, 12 (33%, 95% CI 17–50%) in the posterior fossa and five (14%, 95% CI 2–26%) in middle cranial fossa. The scans showed 13 (1.3%) cysts of other types, see Table 2.

#### Structural vascular abnormalities

There were 19 individuals (1.9%) with aneurysms. Sixteen participants had a singular aneurysm and three had multiple aneurysms (Fig 2). Only five of the 19 persons with aneurysms were men; still the sex-specific prevalence was not significantly different (Table 2). Three participants had cavernous hemangiomas of which two had very small lesions. Four individuals had a deep venous anomaly, and one a Spezler-Martin grade 2 arteriovenous malformation (4.3x2.8x2.6 cm) in the parieto-occiptal lobe (Fig 2). For stenosis and occlusions of cerebral and internal carotid arteries see Table 2.

#### Other developmental variants

Two men had grey matter heterotopia located adjacent to the lateral ventricles, both reported never to have experienced seizures. Two participants had grade 1 Chiari malformation. For megacisterna magna, with and without septum pellucidum see Table 2.

#### Infarctions

44 individuals (4.4%, 95% CI 3–5%), of which 20 were women, had brain infarction(s). There was no difference in the sex specific prevalence for the different types of infarctions. For the anatomical location of the infarctions see Table 2. A total of 16 participants (1.6%, 95% CI 1–2%) had clinical infarctions, and 28 (2.8% 95% CI 2–4%) had silent infarctions (Fig 3). Significantly more of the participants (63.6%, 95% CI 48.8–78.4) had silent compared to clinical infarctions (*p*<0.0001). A moderate effect of age on prevalence of infarction was present; the average age of participants with infarction was 60.5 ± 4.3 years compared to 58.9 ± 4.2 years for infarction free participants (*p* = 0.01, Cohen’s *d* = 0.37). There were significantly more infarctions in left compared to the right cerebral and cerebellar hemispheres (*p*<0.0001). This left hemisphere predominance was present for both silent and clinical infarctions (*p*<0.0001).

#### WMH

In total 91 (9.1%) participants had excessive WMH. There was significantly more women than men (*p* = 0.048) with excessive WMH. A moderate effect of age was observed as individuals with excessive WMH were significantly older with a mean age of 60.5±3.8 years compared to those with age appropriate WMH with a mean age of 58.8±4.2 years (*p*<0.0001, Cohen’s *d* = 0.4). 92.3% of the participants with excessive WMH were unaware of this. Only 7 of the 91 participants (7.7%, 95% CI 2–13%) were informed of the presence of excessive WMH due to previous MRI of the brain as part of diagnostics for various conditions, none of which were related to cerebral ischemia.

#### Microhemorrhages and parenchymal calcifications

Thirteen participants had cerebral microhemorrhages. Eleven had a singular lesion and two multiple lesions (14 and 16 lesions, respectively). None of the individuals with microhemorrhages had contusions, but one had a lacunar infarction combined with excessive WMH. Two subjects had parenchymal calcifications located to globus pallidus and cerebellum, respectively.

#### Tumors

A total of 14 incidental intracranial tumors were detected, giving an overall prevalence of 1.4% (95% CI 1–2%). One glioma was detected in the frontal lobe (Fig 3), histological examination demonstrated a mixed cell glioma, WHO grade 2. There were 10 meningiomas, ranging from a few mm to 29 mm (Fig 3). The meningiomas were equally distributed amongst the ages, but not between the sexes as nine out of ten meningiomas were found in women (*p* = 0.022). There were three pituitary tumors, of which one was previously known. One participant had a vestibular schwannoma.

#### Other intracranial findings related to known acquired brain disorders

One or more traumatic contusions were found in 10 participants (1.0%) who all had experienced moderate head trauma. Three participants had multiple sclerosis, previously clinically acknowledged. One man had known progressive supranuclear palsy. Five participants had undergone brain surgery; one due to chronic subdural hematoma, one temporal pole lobectomy due to epilepsy, one meningioma, one pituitary tumor, and one posterior fossa arachnoid cyst resection.

## Discussion

The HUNT MRI study is the first study to systematically assess the prevalence of incidental intracranial findings, the clinical impact of such findings and the number of false positives on MRI in a representative sample drawn from the general population by accessing participants’ medical history, hospital records, earlier and additional neuroimaging. This is also the first study to consistently report on types of medical follow-up and outcome for those treated surgically or with radiation therapy following discovery of an incidental intracranial findings in a research setting using a prospective design.

### Prevalence of intracranial findings, false positives, follow up and outcome

The overall prevalence of an intracranial finding was 32.7%, or expressed per participant; 24.1% of the participants had one or more type(s) of intracranial finding(s). The majority (83.0%) of the findings were classified as incidental after screening the participants’ medical history, hospital records, previous radiological exams, plus phone interview. Since the current study is the first where type of incidental intracranial finding was verified in this manner, direct comparison with prevalence of incidental intracranial findings in previous studies is difficult. A further complication is that the types of findings reported (i.e. etiologies of findings, and/or entire head versus intracranially), and how incidental findings are classified and assigned clinical impact vary considerably between studies. Likewise, cohort age and characteristics (e.g. hospital-based, volunteers, insurance screening) are highly variable. It is therefore not surprising that the reported prevalence of incidental findings on MRI of the head is variable, from 3% to 34% in previous studies [9–13, 15, 16, 32–37]. The prevalence of incidental intracranial findings in HUNT MRI is in the upper end of the previous reports. Taking into account that most previous studies included extracranial findings, for instance the paranasal sinus, the HUNT MRI prevalences are indeed high. The most comparable results to those in the HUNT MRI cohort are found in Lothian Birth Cohort of 1936 in older adults included at time of their births, and having an MRI of the head around age 72 [13], and the Mind Research Network based on scans from a variety of neurological and psychiatric studies in infants to octogenarians [16]. However, not only intracranial findings were reported in these studies, and the latter included hospital samples. There are several explanations for the high prevalence of incidental findings in HUNT MRI compared to the results in many of the previous reports. First, the HUNT MRI scan protocol is comparable to protocols used for standard diagnostic imaging of brain disorders, while most other studies are based on heterogeneous MRI scans and fewer imaging sequences. There appears to be a higher prevalence of intracranial findings in more recent compared to earlier MRI studies, suggesting that the use of more advanced and comprehensive MRI protocols, often with high-resolution scans, increases the sensitivity for uncovering findings. Second, the reading by two senior neuroradiologists may have contributed to higher number of findings since radiological experts diagnose more lesions and with a higher accuracy than both novice and more experienced residents [38–40]. Interestingly, the Lothian Birth Cohort of 1936 and Mind Research Network studies with the most similar prevalence of findings also had the scans read by (neuro)radiologists while many other studies rely on other professions or especially trained researchers. Third, the HUNT MRI participants are older, both with regard to mean age and age range, than in most previous studies. With age, there is an increase in the prevalence of both cerebrovascular disease and intracranial tumors [12, 15], which constituted the majority of incidental findings and all findings with clinical impact in HUNT MRI. Importantly, HUNT MRI was performed in a documented representative general population as the participants were drawn from the HUNT population representing ~70% of an entire geographical population [17], and further ~73% of those asked agreed to participate in HUNT MRI [21]. Hence, the reported prevalence of the different intracranial findings should be representative of a general population [41]. Indeed, the cohort, MRI protocol and rigorous procedures followed to determine the exact nature of the findings provide considerable strength to the validity and reliability of the present data.

The HUNT MRI data clearly showed that most intracranial findings are incidental, demonstrating that screening of participants’ medical history before MRI scanning will have limited impact on the number of findings uncovered in middle aged and older populations. Furthermore, about 75% of all incidental findings had clinical impact. Acquired and developmental cerebrovascular pathologies made up the bulk of clinically relevant findings (~90%); the rest were intracranial tumors (~10%). In total, ~15% of all HUNT MRI participants or about 1 in 7 middle aged participant from the general population, were followed up clinically. This number is in stark contrast to the number needed to scan of 37 to detect an unexpected abnormality on brain MRI reported in a guideline on management of incidental findings detected in the course of research [42]. The relatively high prevalence of findings with clinical impact underscores the importance of having established good routines for appropriate clinical handling of findings before start of a study to ensure consistency as well as timely and proper follow-up.

Of participants referred to follow-up, most were seen by primary physicians (76.1%) and the remainder clinical specialists (23.9%). Taking into account all the additional neuroimaging performed in the false positives, HUNT MRI resulted in 101 hospital referrals, i.e. ~10% of the cohort received some type of advanced hospital services. In addition, ~10% of the total HUNT MRI cohort was referred to their primary physician. Prevalence of referral in previous studies has usually been categorized into urgent and routine, with urgent referral rates between ~1–4% and non-urgent ~2% [10, 13, 14, 33]. Considerable between study variations with regard to which diagnoses are considered in need of urgent referral makes direct comparison between studies difficult. Nevertheless, in HUNT MRI, both referrals that could be categorized as urgent and as routine were notably more frequent than in all previous reports. The medical benefits of detecting and treating incidental intracranial findings on MRI remain to be firmly established. The underlying tenet of human research is that participants should experience a positive benefit and minimal risk [43]. Based on the literature, it seems likely that cerebral MRI in a research setting can have a positive effect. Interventions aimed at limiting, attenuating or removing risk factors for ischemic cerebro-/cardiovascular diseases have for instance been demonstrated to reduce morbidity and mortality [28, 44, 45]. For the meningiomas, survival has not been shown to be improved by incidental detection [46], but for low-grade gliomas, early and radical surgery significantly improves survival [47]. The management of incidental intracranial aneurysms is a controversial topic in neurosurgical and radiological practice [48]. With an estimated risk of aneurysm rupture of 0.87% per year in this geographic region [49], and taking into account the increased mortality and morbidity connected to subarachnoidal hemorrhage with increasing age plus the success of the aneurysm surgery, the benefit appears to be larger than the risk of treatment of aneurysm(s) in the present study. There was one participant who experienced a permanent neurological deficit, a hemi-quandrantopia, following surgery for an arteriovenous malformation. This was an expected complication based on the type and location of the malformation (Fig 3), and reflects the significant risk related to interventions on arteriovenous malformations [50, 51]. Taken together, these results point to the clinical benefits, even when including surgery, being greater than the harm connected to taking part in a neuroimaging research study for participants with true positive findings. There were, however, ~2% false positives. The vast majority of the false positives were suspected gliomas which turned out to be benign singular white matter lesions after additional MRI examination with contrast agent and MR spectroscopy. This finding illustrates both the difficulty of diagnosing gliomas even with a comprehensive MRI scan protocol, and that we erred on the side of caution. In the perspective of benefit versus harm, participants with false positives may have experienced the uncertainty related to the initial diagnosis as more harmful than helpful. Still, we did not receive feedback on negative experiences related to follow-up imaging to ensure correct diagnosis. The costs related to additional neuroimaging and referral to primary physicians and clinical specialists were not calculated, and will vary depending on the health care system. None-the-less, the number of incidental intracranial findings and follow up of these in HUNT MRI clearly elucidates the need for good procedures for appropriate follow-up to ensure that participants receives the benefit of participating in a research study.

### Types of intracranial findings

The present data clearly show that there are numerous different types of intracranial findings in a general population, with most findings being very rare. Indeed, only arachnoid cysts, aneurysms, infarctions and excessive WMH were present in more than 1% of the participants.

#### Cysts

The most common incidental finding was arachnoid cysts with a prevalence of 3.6%. This prevalence is higher than the 0.6–2.6% in previous MRI studies [11, 12, 35, 52]. The higher number may be due to the use of higher resolution MRI scans, review by two highly experienced neuroradiologists, and no lower limit of cyst size to be reported. The current figure of 3.6% may hence represent an accurate estimate of the prevalence of arachnoid cysts per se. There were also several other types of cysts identified in the HUNT MRI cohort, but except for ependymal cysts, most were very rare, with lower prevalence than previously reported. For instance, the prevalence of pineal gland cysts was 100–10 000 times lower than in healthy control and patients investigated with MRI [32, 53]. Likewise, the prevalence of Rathke’s cleft cysts was very low compared to autopsy reports [54, 55], and an MRI study reporting 85% prevalence [56]. Since there is no reason to believe that the MRI scan protocol in this study lacked the resolution or contrast needed to uncover different types of cysts, it seems likely that cyst prevalence is highly dependent on population scanned.

#### Cerebrovascular pathologies

The most frequent incidental findings with clinical impact were related to various developmental and acquired cerebrovascular pathologies in the current study. Excessive WMH were present in ~9% of the HUNT MRI cohort, which is in the high end compared to the 4–7% reported previously [12, 57], but lower than in the slightly older Lothian birth cohort of 1936 [13]. Age is significantly associated with WMH [15, 58, 59], which was also demonstrated in the present study, and explains some of the variability between different studies. Hypertension is another risk factor associated with increased load of WMH [60]. Interestingly, women, but not men, in the HUNT MRI cohort had a significantly reduced systolic blood pressure compare with those declining participation [21]. Still, excessive WMH were significantly more prevalent in women than men. This finding points to sexual dimorphism in the development of WMH.

Silent infarctions were more prevalent than clinical infarctions, present in ~64% of the individuals with infarctions. The preponderance of silent over clinical infarctions concurs with previous results based on MRI. Still, only 2.8% of the HUNT MRI participants had silent infarctions, which is considerably lower than the ~4–28% reported in previous MRI studies [61–64]. Also the prevalence of clinical infarctions was lower in HUNT MRI than in previous clinical reports from the same geographical region [65] and internationally [66]. One might speculate that the somewhat better cardiovascular risk profile, lower weight and higher education in the HUNT MRI may have reduced the overall number of brain infarctions, but in light of the high prevalence of WMH this explanation is not entirely satisfactory.

Cerebral microhemorrhages occurred in 1.3% of the participants, and were only considered to be clinically significant in one subject. The overall prevalence of microhemorrhages was similar to that in the Lothian Birth cohort [13], but considerably lower than the ~3–8% reported in other similarly aged, healthy controls also based on 2D T2* gradient echo scans [67–72]. Microhemorrhages are associated with risk factors for cardiovascular disease, namely hypertension, diabetes mellitus and inflammation, and known cerebrovascular disease [72, 73]. As mentioned above, the low prevalence of microhemorrhages could be due to the fact that the HUNT MRI cohort was relatively healthy. However, the similar low prevalence in the Lothian Birth cohort and much higher prevalence in other cohorts, suggest that regional and/or population dependent factors are important for the prevalence of microhemorrhages.

The prevalence of aneurysms was ~2%, which is similar to that in the Rotterdam scan study, the only other general population-based study on aneurysm prevalence [15]. The 2% aneurysm prevalence is low compared to meta-analysis in selected groups, probably reflecting both selection bias and the substantial population differences in aneurysm prevalence [31, 74].

#### Intracranial tumors

The prevalence of 1.4% intracranial tumors and the preponderance of extra- over intra-axial tumors (13:1) in the HUNT MRI cohort are in agreement with previous reports in middle aged and older subjects [12, 13, 15]. The principal intracranial tumor was meningioma (1% of the participants) concurring with results in similar age groups based on MRI and autopsy [13, 15, 75]. Nine out of 10 meningiomas were found in women. That meningiomas are more common in women is well known, although the male: female ratio varies notably between studies [36, 75], with the present result somewhere in the middle. There was one glioma, giving an incidence of 0.1% of finding a glioma in line with clinical data in Caucasians [76–78].

### Limitations

The MRI scan protocol has significant impact on type of lesions which can be visualized. The HUNT MRI protocol allows for detection of the most common brain pathologies. Still, implementing susceptibility weighted imaging (SWI) instead of the gradient echo T2*scan would have led to greater sensitivity for microhemorrhages, deep vessel anomalies and calcifications. Moreover, even though the types of findings recorded were comprehensive the list is not exhaustive, as we did not evaluate all types of normal variations, i.e. related to vessel morphometry, or perform semi-qualitative assessment of different types of cerebral atrophy. However, none of the HUNT MRI participants have been diagnosed with mild cognitive impairment or dementia at time of manuscript submission. Furthermore, as we did not have access to medical and radiological records unless a finding was uncovered during reading of the HUNT MRI data, we were unable to assess false negatives, and thus the sensitivity of MRI for use in a general population could unfortunately not be evaluated.

## Conclusion

In the HUNT MRI cohort in subjects between 50 and 66 years of age, ~17% of all participants were followed up by their primary physician, a clinical specialist or additional neuroimaging, demonstrating the need for setting up appropriate routines before a neuroimaging research study commences. About 90% of the incidental findings with clinical impact were related to the different developmental and acquired cerebrovascular pathologies (WMH, infarctions, aneurysms and other vessel malformations, micorhemorrhage, vessel occlusion and stenosis). Unrecognized cerebral small vessel disease (i.e. excessive WMH) was the most frequent of all incidental findings, and silent infarctions were more common than clinical infarctions underscoring a large potential for different types of measures aimed at preventing and/or ameliorating unrecognized arteriosclerotic disease to maintain brain health.
